# Altered Expression of Mitochondrial NAD^+^ Carriers Influences Yeast Chronological Lifespan by Modulating Cytosolic and Mitochondrial Metabolism

**DOI:** 10.3389/fgene.2018.00676

**Published:** 2018-12-19

**Authors:** Ivan Orlandi, Giulia Stamerra, Marina Vai

**Affiliations:** ^1^SYSBIO Centre for Systems Biology, Milan, Italy; ^2^Dipartimento di Biotecnologie e Bioscienze, Università di Milano-Bicocca, Milan, Italy

**Keywords:** NAD^+^, chronological lifespan, Ndt1, Ndt2, *Saccharomyces cerevisiae*

## Abstract

Nicotinamide adenine dinucleotide (NAD^+^) represents an essential cofactor in sustaining cellular bioenergetics and maintaining cellular fitness, and has emerged as a therapeutic target to counteract aging and age-related diseases. Besides NAD^+^ involvement in multiple redox reactions, it is also required as co-substrate for the activity of Sirtuins, a family of evolutionary conserved NAD^+^-dependent deacetylases that regulate both metabolism and aging. The founding member of this family is Sir2 of *Saccharomyces cerevisiae*, a well-established model system for studying aging of post-mitotic mammalian cells. In this context, it refers to chronological aging, in which the chronological lifespan (CLS) is measured. In this paper, we investigated the effects of changes in the cellular content of NAD^+^ on CLS by altering the expression of mitochondrial NAD^+^ carriers, namely Ndt1 and Ndt2. We found that the deletion or overexpression of these carriers alters the intracellular levels of NAD^+^ with opposite outcomes on CLS. In particular, lack of both carriers decreases NAD^+^ content and extends CLS, whereas *NDT1* overexpression increases NAD^+^ content and reduces CLS. This correlates with opposite cytosolic and mitochondrial metabolic assets shown by the two types of mutants. In the former, an increase in the efficiency of oxidative phosphorylation is observed together with an enhancement of a pro-longevity anabolic metabolism toward gluconeogenesis and trehalose storage. On the contrary, *NDT1* overexpression brings about on the one hand, a decrease in the respiratory efficiency generating harmful superoxide anions, and on the other, a decrease in gluconeogenesis and trehalose stores: all this is reflected into a time-dependent loss of mitochondrial functionality during chronological aging.

## Introduction

Significant progress has been made in elucidating fundamental processes such as human aging/ longevity as a result of studies performed in the budding yeast *Saccharomyces cerevisiae*. In this single-celled yeast, replicative aging and chronological aging are two complementary models that are used to simulate cellular aging of mitotically active and post-mitotic mammalian cells, respectively (MacLean et al., 2001; Longo and Kennedy, 2006; Longo et al., 2012). The former cell type is exemplified by fibroblasts and the latter by myocytes.

In the presence of nutrients, *S.cerevisiae* divides asymmetrically (budding) resulting in a large mother cell and a smaller daughter (bud). In this context, the replicative lifespan (RLS), namely the number of buds generated by a mother cell before senescence, indicates the reproductive potential of individual yeast cells (Steinkraus et al., 2008). The chronological lifespan (CLS), instead, refers to the rate of post-mitotic survival of a non-dividing quiescent yeast culture; viability is assessed by measuring the percentage of cells able to resume growth and form a colony after transfer from the depleted medium to the rich fresh one (Fabrizio and Longo, 2007). In a standard CLS experiment, yeast cells are grown in synthetic media with 2% glucose. When glucose becomes limiting, the diauxic shift occurs and cells shift from glucose-driven fermentation to ethanol-driven respiration. This shift determines a metabolic reprogramming, the outcomes of which influence the CLS. Afterwards cell proliferation stops and the yeast culture enters a quiescent stationary phase (Gray et al., 2004; Wanichthanarak et al., 2015). CLS is determined starting 72 h after the diauxic shift (Fabrizio and Longo, 2007).

The signaling pathways and regulators controlling RLS and CLS are evolutionary conserved (Fontana et al., 2010; Swinnen et al., 2014; Bitto et al., 2015; Baccolo et al., 2018). In particular, nicotinamide adenine dinucleotide (NAD^+^) homeostasis has emerged as a critical element in the regulation of aging/longevity (Imai, 2010, 2016) and accumulating evidence suggests that a reduction of NAD^+^ levels in diverse organisms contributes to the development of age-associated metabolic decline (Imai and Guarente, 2014, 2016; Verdin, 2015). Indeed, in addition to its central role in cellular metabolism participating as essential coenzyme in many redox reactions, NAD^+^ is absolutely required as a co-substrate by Sirtuins, a family of NAD^+^-dependent deacetylases, the founding member of which is Sir2 of *S.cerevisiae* (Houtkooper et al., 2012; Imai and Guarente, 2014). In mammals, there are seven Sirtuin isoforms (SIRT1-7) and among them SIRT1 is a key component of the systemic regulatory network called “the NAD world,” a comprehensive concept that connects NAD^+^ metabolism and aging/longevity control in mammals (Imai, 2009, 2010, 2016). The nutrient-sensing SIRT1 is the closest mammalian ortholog of Sir2 (Frye, 2000). Sir2 activity is involved in both replicative and chronological aging: in the former Sir2 extends RLS (Kaeberlein et al., 1999; Imai et al., 2000), whilst in the latter it has a pro-aging role (Fabrizio et al., 2005; Smith et al., 2007; Casatta et al., 2013; Orlandi et al., 2017a).

The other key component of the NAD world is represented by NAD^+^ biosynthesis (Imai, 2009, 2010). From yeast to mammalian cells, NAD^+^ synthesis occurs either *de novo* from L- tryptophan or through *salvage* pathway(s) from its precursors, namely nicotinamide riboside, nicotinic acid, and its amide form, nicotinamide (Bogan and Brenner, 2008; Canto et al., 2015). Cells mainly rely on the *salvage* pathway(s) for the correct maintenance of NAD^+^ levels and it has been observed that the supplementation of NAD^+^ precursors is sufficient to attenuate several metabolic defects common to the aging process (Johnson and Imai, 2018; Mitchell et al., 2018; Rajman et al., 2018). However, NAD^+^ levels, as well as those of its precursors, are different depending on the type of tissue and cellular compartment (Dolle et al., 2010; Houtkooper et al., 2010; Cambronne et al., 2016) and it remains unclear in which cellular compartment(s) NAD^+^ decrease can be relevant to aging. This has increased the interest on the role, on the one hand, of inter-tissue communications (Imai, 2016) and, on the other hand, of the relative subcellular localization of NAD^+^ and its precursors during the aging process (Koch-Nolte et al., 2011; Rajman et al., 2018).

In yeast, NAD^+^ is synthesized in the cytosol and can be imported across the inner mitochondrial membrane by two specific mitochondrial NAD^+^ carriers, namely Ndt1 and Ndt2, which share 70% homology (Todisco et al., 2006). The physiological effects linked to an *NDT1* and *NDT2* double deletion and to the overexpression of *NDT1*, which encodes the main isoform of the NAD^+^ transporter (Todisco et al., 2006), have been examined on cells growing with an oxidative or respiro-fermentative metabolism in batch and glucose-limited chemostat cultures (Agrimi et al., 2011).

Here, we show that during chronological aging an altered expression of the specific mitochondrial NAD^+^ carriers deeply influences the metabolic reprogramming that enables cells to acquire features required to maintain viability during chronological aging. In particular, lack of *NDT1* and *NDT2* extends CLS, whereas *NDT1* overexpression determines a CLS reduction. This opposite effect on CLS correlates with opposite metabolic features displayed by the two mutants.

## Materials and Methods

### Yeast Strains, Growth Conditions and CLS Determination

The *ndt1*Δ*ntd2*Δ strain and the strain overexpressing *NDT1* (*NDT1*-over strain) were constructed in a previous work (Agrimi et al., 2011) and were derivatives of CEN.PK113-7D (*MAT*a, *MAL*2-8c, *SUC*2). A null mutant *ndt1*Δ*ntd2*Δ (*ndt1*Δ::*URA3 ntd2*Δ::*KlLEU2*) was generated by PCR-based methods in a W303-1A background (*MAT*a *ade2-1 his3-11,15 leu2-3,112 trp1-1 ura3-1 can1-100*). The accurancy of gene replacements was verified by PCR with flanking and internal primers. Cells were grown in batches at 30°C in minimal medium (Difco Yeast Nitrogen Base without amino acids, 6.7 g/L) with 2% w/v glucose. Auxotrophies were compensated for with supplements added in excess (Orlandi et al., 2014). Cell number and cellular volumes were determined using a Coulter Counter-Particle Count and Size Analyser (Vanoni et al., 1983). Duplication time (Td) was obtained by linear regression of the cell number increase over time on a semi-logarithmic plot. For CLS experiments, cells were grown in 2% glucose and the extracellular concentration of glucose and ethanol were measured in medium samples collected at different time-points in order to define the growth profile [exponential phase, diauxic shift (Day 0), post-diauxic phase and stationary phase of the cultures] (Orlandi et al., 2013). CLS was measured according to (Fabrizio et al., 2005) by counting colony-forming units (CFU) starting with 72 h (Day 3, first age-point) after Day 0. The number of CFU on Day 3 was considered the initial survival (100%).

### Isolation of Mitochondria

Mitochondria were prepared from chronologically aging cells essentially as described by Meisinger et al. (2006) with minor modifications. At each time-point, 10^9^ cells were collected by centrifugation and spheroplasts were obtained by digestion with Zymolyase 20T. Then, spheroplasts were homogenized by 20 strokes using a Dounce homogenizer and mitochondria collected after differential centrifugation (Meisinger et al., 2006). Fresh crude mitochondrial pellets were used for measurements of NAD^+^, NADH, and protein contents.

### Metabolite Measurements and Enzymatic Assays

At designated time-points, aliquots of the yeast cultures were centrifuged, and both pellets (washed twice) and supernatants were collected and frozen at −80°C until used. Rapid sampling for intracellular metabolite measurements was performed as previously described (Orlandi et al., 2014). The concentrations of glucose, ethanol, citrate, succinate, and malate were determined using enzymatic assays (K-HKGLU, K-ETOH, K-SUCC, K-CITR, and K-LMALR kits from Megazyme).

To measure NADH and NAD^+^ contents, alkali, and acid extractions were performed essentially as described (Lin et al., 2001), except that before incubation of both the alkali extract and the acid one at for 30 min, an additional step was performed in order to improve cells lysis. Alkali or acid-washed glass beads were added to the two types of extracts and cells broken by vortexing (3 cycles of 1 min, interspersed with cooling on ice). NAD^+^ and NADH concentrations were determined using the EnzyChrom^TM^ NAD^+^/NADH assay kit (BioAssay Systems). The rate of dye formation (formazan) at 565 nm correlates with the level of pyridine nucleotides. Duplicate reactions were performed in multi-well plates or in cuvettes. Different amounts of each sample were used in cycling reactions to obtain values within the linear portion of a standard curve that was prepared every time.

Immediately after preparation of cell-free extracts (Orlandi et al., 2014), the activities of cytosolic and mitochondrial aldehyde dehydrogenase (Ald) were assayed according to Aranda and del Olmo (2003), of phosphoenolpyruvate carboxykinase (Pck1) and isocitrate lyase (Icl1) as described in de Jong-Gubbels et al. (1995). Total protein concentration was estimated using the BCA^TM^ Protein Assay Kit (Pierce).

### Fluorescence Microscopy

Dihydroethidium (DHE, Sigma-Aldrich) staining was performed as reported in Madeo et al. (1999) to detect superoxide anion (${\text{O}}_{2}^{-}$). A Nikon Eclipse E600 fluorescence microscope equipped with a Nikon Digital Sight DS Qi1 camera was used. Digital images were acquired and processed using Nikon software NIS-Elements.

### Estimation of Oxygen Consumption Rates and Index of Respiratory Competence

The basal oxygen consumption of intact cells was measured at 30°C using a “Clark-type” oxygen electrode (Oxygraph System, Hansatech Instruments, Nortfolk, UK) as reported (Orlandi et al., 2013). The non-phosphorylating respiration and the maximal/uncoupled respiratory capacity were measured in the presence of 37.5 mM triethyltin bromide (TET, Sigma-Aldrich) and 10 μM of the uncoupler carbonyl cyanide 3-chlorophenylhydrazone (CCCP, Sigma-Aldrich), respectively (Orlandi et al., 2017a). The addition of 2 M antimycin A (Sigma-Aldrich) accounted for non-mitochondrial oxygen consumption. Respiratory rates for the basal oxygen consumption (J_R_), the maximal/uncoupled oxygen consumption (J_MAX_) and the non-phosphorylating oxygen consumption (J_TET_) were determined from the slope of a plot of O_2_ concentration against time, divided by the cellular concentration.

Index of respiratory competence (IRC) was measured according to Parrella and Longo (2008) by plating identical cell samples on YEP (1% w/v yeast extract, 2% w/v bacto peptone)/2% glucose (YEPD) plates and on rich medium/3% glycerol (YEPG) plates. IRC was calculated as colonies on YEPG divided by colonies on YEPD times 100%.

### Statistical Analysis of Data

All values are presented as the mean of three independent experiments ± Standard Deviation (SD). Three technical replicates were analyzed in each independent experiment. Statistical significance was assessed by one-way ANOVA test. The level of statistical significance was set at a *P* value of ≤ 0.05.

## Results and Discussion

### Altered Expression of the Specific Mitochondrial NAD^+^ Carriers Affects CLS

Due to the importance of NAD^+^ homeostasis in the aging process from yeast to humans (Baccolo et al., 2018; Rajman et al., 2018; Yaku et al., 2018), we wished to test whether changes in the cellular content of this dinucleotide would cause any effects on CLS. To this end we chose to use the *ndt1*Δ*ndt2*Δ and *NDT1*-over mutant strains: the former lacking the two mitochondrial NAD^+^ carriers, Ndt1 and Ndt2, identified so far and the latter overexpressing Ndt1, which is the main isoform of the carrier (Todisco et al., 2006; Agrimi et al., 2011). These strains have been previously characterized as far as NAD content is concerned (Agrimi et al., 2011). In particular, under a fully respiratory metabolism such as growth on ethanol, Ndt1 overexpression determined an increase in cellular and mitochondrial NAD^+^ levels without affecting growth. On the contrary, on ethanol the *ndt1*Δ*ndt2*Δ mutant displayed a lower cellular and mitochondrial NAD^+^ content and a decrease in the growth rate (Agrimi et al., 2011). Here, an *ndt1*Δ*ndt2*Δ double mutant generated in the W303-1A background was also included. Indeed, the W303-1A strain is commonly used in chronological aging research due to its robust respiratory capacity (Ocampo et al., 2012).

**Figure 1:**
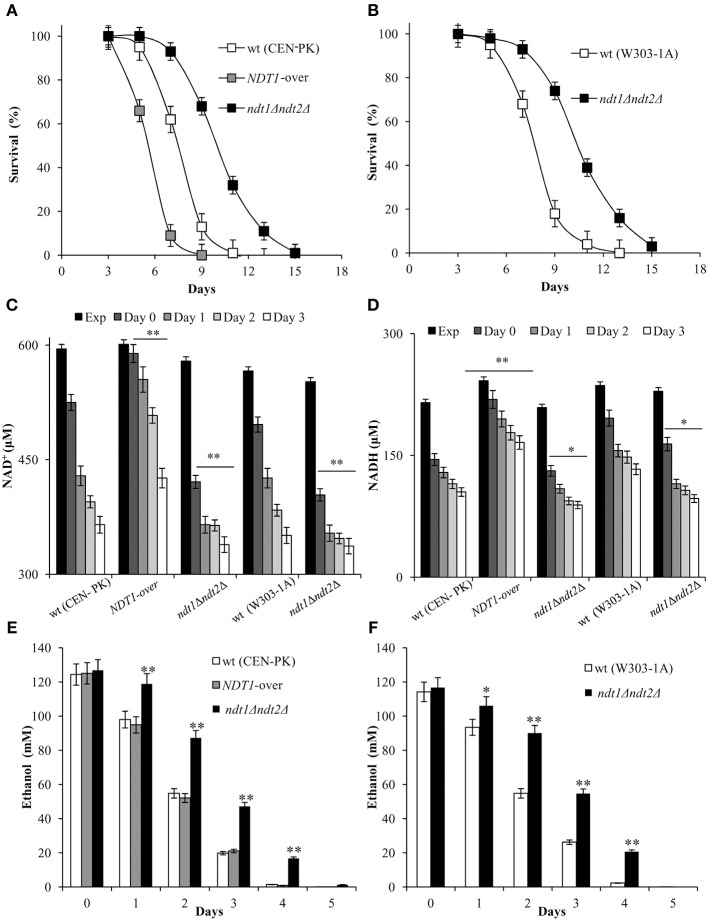
The *NDT1*-over mutant and the *ndt1*Δ*ndt2*Δ one have a short-lived and a long-lived phenotype, respectively. The indicated strains were grown in minimal medium/2% glucose and the required supplements in excess (see section Materials and Methods) and followed up to stationary phase. **(A,B)** CLS was determined by assessing clonogenicity on YEPD plates. 72 h after the diauxic shift (Day 3) was considered the first age-point (100% survival). Day 0, diauxic shift. In parallel, intracellular NAD^+^ **(C)**, NADH **(D)** and extracellular ethanol **(E,F)** concentrations were determined at the indicated time-points. Exp, exponential growth phase. All data refer to mean values of three independent experiments with three technical replicates each. Standard deviation (SD) is indicated. Statistical significance as assessed by one-way ANOVA test is indicated (^*^P ≤ 0.05 and ^**^P ≤ 0.01).

Initially, in the context of a standard CLS experiment (Fabrizio and Longo, 2007), we measured CLS and NAD content. As shown in Figure 1A, Ndt1 overexpression significantly reduced CLS, whilst the strain devoid of the two mitochondrial NAD^+^ carriers lived longer than the prototrophic wild type (wt) CEN.PK 113-7D. The same long-lived phenotype was observed in the auxotrophic background W303-1A (Figure 1B) indicating that the different composition of amino acids in the medium does not influence the results. Measurements of intracellular NAD^+^ and NADH contents indicated that in the wt, they decreased progressively after the diauxic shift (Figures 1C,D). We calculated values of NAD^+^ and NADH estimating cell size with a Coulter Counter-Particle Count and Size Analyser: cell size that changes according to the yeast strain and the growth phase of the cell cycle. If we assume a yeast cell size of 70 μm^3^ (Sherman, 2002) our measurements of 595 μM NAD^+^ (Figure 1C) and 215 μM NADH for CEN.PK 113-7D in exponential phase (Figure 1D) correspond to 1.42 mM NAD^+^ and 0.82 mM NADH, in reasonable agreement with values of previous reports (Lin et al., 2004). In the context of the CLS experimental set-up, as the diauxic shift occurs and cells utilize the excreted fermentation by-product, ethanol, in the *ndt1*Δ*ndt2*Δ mutant, and in the *NDT1*-over one NAD^+^ and NADH levels decreased, but both remained constantly lower in the *ndt1*Δ*ndt2*Δ mutant and higher in the *NDT1*-over mutant than those measured in the wt (Figures 1C,D). This opposite trend of the dinucleotide contents observed in the two different types of mutants is in line with that detected during exponential growth on ethanol (Agrimi et al., 2011).

**Figure 2:**
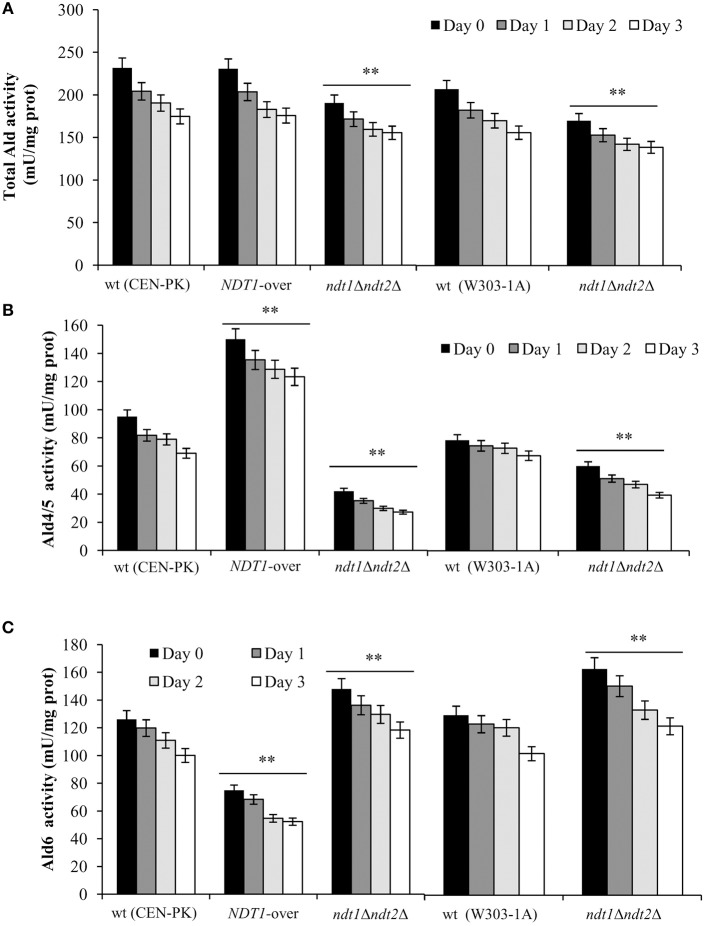
Altered expression of mitochondrial NAD^+^ carriers influences Ald enzymatic activities. In the context of CLS experiments of Figure 1, total Ald **(A)**, Ald4/5 **(B)**, and Ald6 **(C)** enzymatic activities were measured at the indicated time-points. Day 0, diauxic shift. All data are the mean ± SD obtained from three independent experiments with three technical replicates each. Statistical significance as in Figure 1 (^**^P ≤ 0.01).

**Figure 3:**
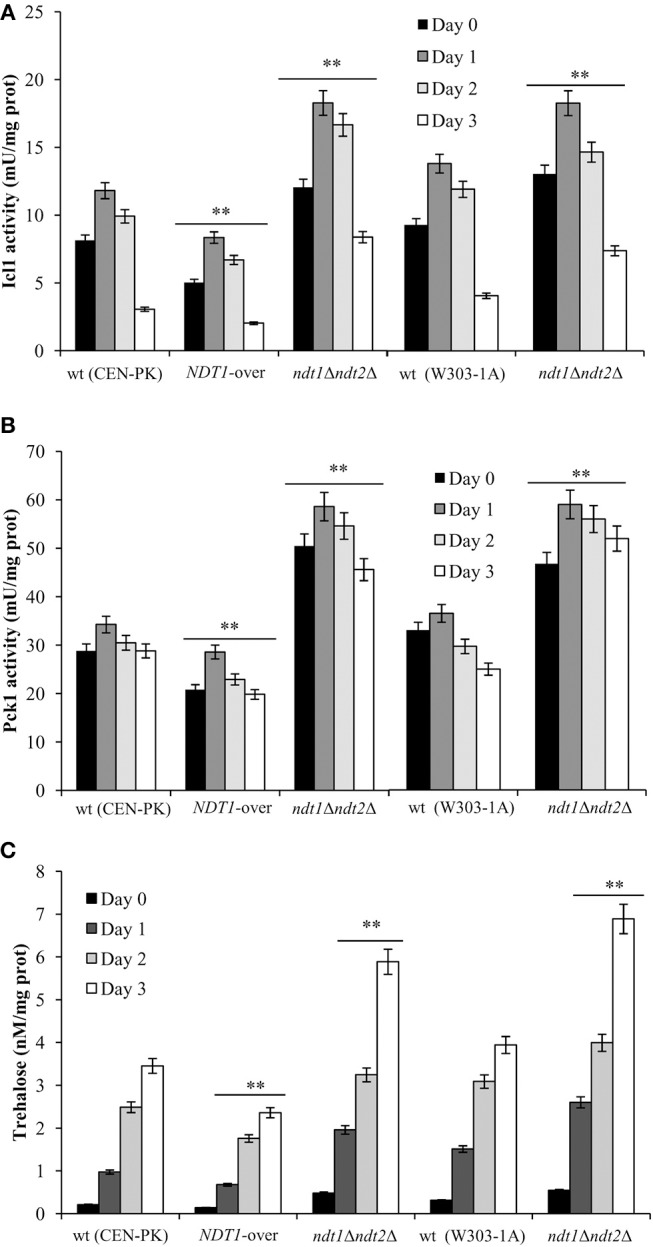
Altered expression of mitochondrial NAD^+^ carriers influences gluconeogenesis and threalose levels. Bar charts of Icl1 **(A)** and Pck1 **(B)** enzymatic activities and intracellular trehalose concentrations **(C)** determined for the indicated strains grown as in Figure 1. Day 0, diauxic shift. All data refer to mean values of three independent experiments with three technical replicates each. SD is indicated (^**^P ≤ 0.01).

It is well known that NAD^+^ is an essential coenzyme for oxidoreductases of both cytosolic and mitochondrial redox reactions, many of which are involved in the metabolic remodeling that takes place at the diauxic shift. Indeed, at the diauxic shift carbon metabolism shifts from fermentation to mitochondrial respiration and gluconeogenesis allowing cells to be better primed for survival during chronological aging. Thus, we analyzed the metabolic features of the short-lived *NDT1*-over strain and those of the long-lived *ndt1*Δ*ndt2*Δ one. Since the respiration-based metabolism is due to the utilization of ethanol, we initially measured the consumption of this C2 compound. At the diauxic shift (Day 0), the maximal amount of the extracellular ethanol was not affected either by the lack of Ndt1 and Ndt2 or by the Ndt1 overexpression (Figures 1E,F). Differently, during the post-diauxic phase in the *ndt1*Δ*ndt2*Δ mutant ethanol decreased more slowly (Figures 1C,D). This is indicative of an impairment in ethanol utilization in line with the slow growth rate on medium containing ethanol as carbon source (Agrimi et al., 2011). Consequently, starting from Day 0, we determined the enzymatic activities of the acetaldehyde dehydrogenases (Alds). These enzymes are implicated in the ethanol utilization: they oxidize the acetaldehyde generated from ethanol oxidation producing acetate, which is subsequently converted to acetyl-CoA. In addition, Alds require NAD^+^ or NADP^+^. No difference was detected between the wt and the *NDT1*-over strain in the total Ald activity levels (Figure 2A). On the contrary, in the *ndt1*Δ*ndt2*Δ strain a significant decrease was observed (Figure 2A) consistent with the reduced ethanol utilization. Notably, interesting results were obtained by measuring the different isoforms of Alds, namely the mitochondrial Ald4/5, and the primary cytosolic counterpart Ald6 (Saint-Prix et al., 2004). Indeed, the activity levels of Ald4/5 were higher and those of Ald6 lower in the *NDT1*-over strain compared with the wt ones, whilst in the *ndt1*Δ*ndt2*Δ strain the Ald6 activity prevailed (Figures 2B,C). Since, alterations of the mitochondrial NAD^+^ transport are accompanied by a different prevalent subcellular localization of Ald enzymatic activities (Table 1), it is reasonable to speculate that in the two different mutants the metabolic pathways that are fed by mitochondrial or cytosolic acetate/acetyl-CoA could be affected. In this context, we initially measured the enzymatic activity of one of the unique enzymes of the glyoxylate shunt, such as isocitrate lyase (Icl1), and that of phosphoenolpyruvate carboxykinase (Pck1), which catalyzes the rate-limiting step in gluconeogenesis. Indeed, starting from the diauxic shift, the glyoxylate shunt becomes operative. It is an anaplerotic device of the TCA cycle, is fed by the cytosolic acetyl-CoA and is the sole possible provider for the Pck1 substrate, namely oxaloacetate (Lee et al., 2011). In the *NDT1*-over strain a decrease in the enzymatic activities of Icl1 and Pck1 was observed, whilst in the *ndt1*Δ*ndt2*Δ mutant both activities strongly increased (Figures 3A,B). Since glucose-6-phosphate produced by gluconeogenesis is used for the synthesis of threalose during the post-diauxic phase, we also examined the accumulation of this disaccharide, the intracellular stores of which are advantageous for survival during chronological aging (Shi et al., 2010). In the *NDT1*-over strain a reduction in trehalose levels took place (Figure 3C), consistent with the decrease of the Pck1 activity. On the contrary, the *ndt1*Δ*ndt2*Δ cells accumulated more trehalose (Figure 3C), consistent with the increase of the Pck1 activity. Taken together, these results indicate that the lack of the two mitochondrial NAD^+^ carriers elicits an enhancement along the cytosolic Ald6/glyoxylate/gluconeogenesis axis, whereas Ndt1 overexpression elicits a down-regulation.

**Table 1:** Effects of altered expression of mitochondrial NAD^+^ carriers on the enzymatic activity of Ald isoforms.

	**Day 0**	**Day 1**	**Day 2**	**Day 3**
**wt (CEN-PK 113-7D)**				
Total Ald	227.8 ± 8.7	202.2 ± 3.9	188.3 ± 4.1	170.8 ± 6.9
% Ald6	57	59	58	59
% Ald4/5	43	41	42	41
***NDT1*****-OVER**				
Total Ald	231.3 ± 4.2	203.6 ± 6.5	182.1 ± 2.7	176.2 ± 1.4
% Ald6	32^**^	33^**^	30^**^	29^**^
% Ald4/5	68^**^	67^**^	70^**^	71^**^
***ndt1*****Δ*****ndt2Δ***				
Total Ald	190.4^**^± 5.6	171.6^**^± 7.2	159.5^**^± 4.3	155.6^**^± 8.1
% Ald6	78^**^	79^**^	81^**^	77^**^
% Ald4/5	22^**^	21^**^	19^**^	23^**^
**wt (W303-1A)**				
Total Ald	202.9 ± 6.4	185.6 ± 3.8	172.4 ± 7.6	156.2 ± 4.7
% Ald6	59	61	55	63
% Ald4/5	41	39	45	37
***ndt1Δndt2Δ***				
Total Ald	174.3^**^± 2.9	157.8^**^± 8.3	146.1^**^± 6.1	128.9^**^± 7.7
% Ald6	83^**^	78^**^	84^**^	79^**^
% Ald4/5	17^**^	22^**^	16^**^	21^**^

For each time-point total Ald, Ald6, and Ald4/5 enzymatic activities were determined as in Figure 2 and the percentage of the different Ald isoforms was calculated. Day 0, diauxic shift. Data refer to mean values determined in three independent experiments with three technical replicates each. SD is indicated. Values obtained for wt strains were used as reference for comparisons with NDT1-over and ndt1Δ ndt2Δ cells. (

** *P ≤ 0.01, one-way ANOVA test)*.

**Figure 4:**
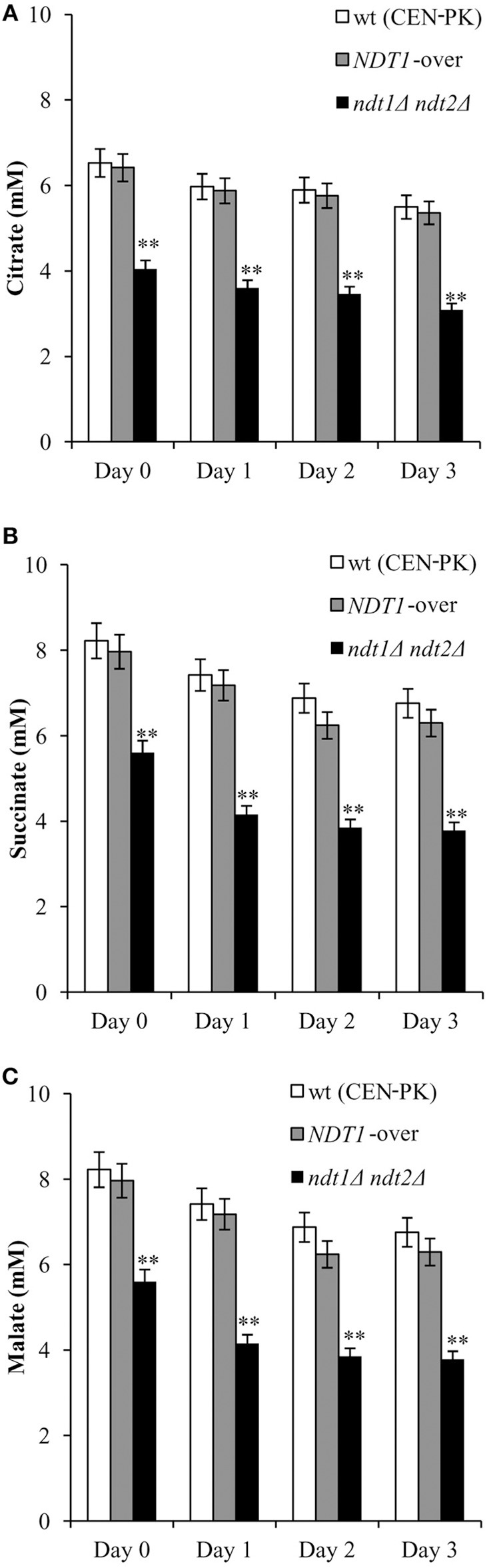
The *ndt1*Δ*ndt2*Δ mutant displays decreased levels of TCA intermediates. The indicated strains were grown as in Figure 1 and intracellular concentrations of citrate **(A)**, succinate **(B)** and malate **(C)** were measured. Day 0, diauxic shift. Bar charts show the mean values determined in three independent experiments with three technical replicates each. SD is indicated (^**^P ≤ 0.01).

Following on, since the TCA cycle is fed by the mitochondrial acetyl-CoA, we assessed the levels of some of its intermediates, such as citrate, malate, and succinate. Starting from the diauxic shift, the levels of these C4 dicarboxylic acids in the *NDT1*-over strain mirrored those measured in the wt (Figure 4). On the contrary, in the *ndt1*Δ*ndt2*Δ mutant all these metabolites significantly decreased (Figure 4) suggesting an impairment in the TCA cycle.

### The *ndt1Δndt2Δ* Mutant Preserves Functional Mitochondria During Chronological Aging

**Figure 5:**
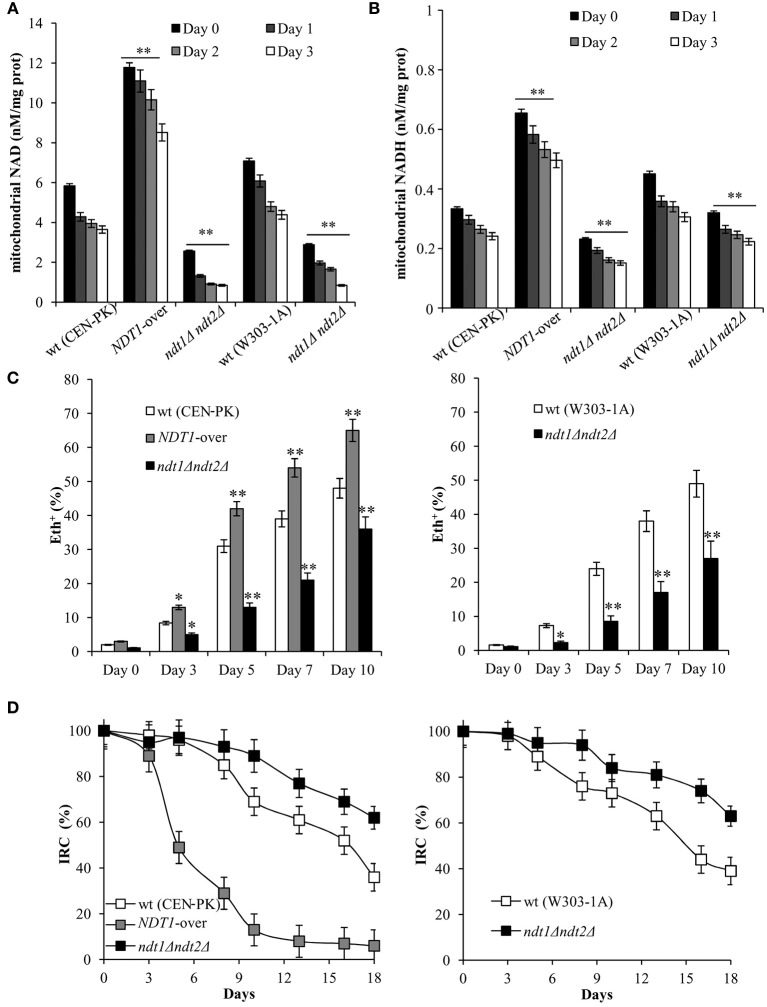
Lack of Ndt1 and Ndt2 has a positive effect on mitochondrial functionality during chronological aging. Cells were grown as in Figure 1. Mitochondrial NAD^+^ **(A)** and NADH **(B)** contents were determined at the indicated time-points. Day 0, diauxic shift. Bar charts show the mean values determined in three independent experiments with three technical replicates each. SD is indicated (^**^P ≤ 0.01). **(C)** Bar charts of the percentage of fluorescent/superoxide positive cells assessed by the superoxide-driven conversion of non-fluorescent dihydroethidium into fluorescent ethidium (Eth). Day 0, diauxic shift. About 1,000 cells for each sample (three technical replicates) in three independent experiments were examined. SD is indicated (^*^P ≤ 0.05 and ^**^P ≤ 0.01). **(D)** Starting from the diauxic shift (Day 0), at indicated time-points aliquots of wt, *NDT1*-over, and *ndt1*Δ*ndt2*Δ cultures were serially diluted and plated onto YEPD and YEPG plates in order to determine the index of respiratory competence (IRC). SD is indicated.

Considering that during respiration the TCA cycle provides the electron transport chain (ETC) with reducing equivalents through redox reactions and that respiration affects the CLS (Bonawitz et al., 2006; Ocampo et al., 2012; Baccolo et al., 2018), we measured next the respiratory activity in the *NDT1*-over and *ndt1*Δ*ndt2*Δ strains. During the exponential phase, the respiratory parameters for the *NDT1*-over and *ndt1*Δ*ndt2*Δ strains were very similar to those of the wt (Table 2) in good agreement with (Agrimi et al., 2011). Differences were observed starting from the diauxic shift (respiratory metabolism). Indeed, in the double deleted mutant, basal oxygen consumption (J_R_) was lower than the wt one (Table 2). This can be ascribed to a depletion/limitation of reducing equivalents since in the presence of the uncoupler CCCP, which dissipates the proton gradient across the mitochondrial membrane, the maximal oxygen consumption rate (J_MAX_) of the *ndt1*Δ*ndt2*Δ cells was always lower than that of the wt (Table 2). Interestingly, the *ndt1*Δ*ndt2*Δ cells displayed a non-phosphorylating respiration (J_TET_) strongly reduced compared with that of the wt, the levels of which increased as a function of time in culture (Table 2) as expected (Orlandi et al., 2017a,b). As a consequence, in the double deleted mutant the net respiration, which estimates the coupled respiration, was close to the wt one (Table 2) indicating that, despite a reduced J_R_, during the post-diauxic phase the *ndt1*Δ*ndt2*Δ strain has a better coupling between electron transport and ATP synthesis. On the contrary, the *NDT1*-over strain had a J_R_ similar to the wt one and a J_MAX_ higher (Table 2). Nevertheless, in this strain the net respiration was lower due to a J_TET_ significantly higher than that of the wt (Table 2) indicative of an increase of uncoupled respiration. These differences in both the level and in the state of the respiration of the two mutants were accompanied by differences in mitochondrial NAD^+^ and NADH contents: in the *ndt1*Δ*ndt2*Δ mutant and in the *NDT1*-over one a decrease and an increase of NAD^+^ and NADH contents, respectively, were observed compared with those of the wt (Figures 5A,B). This opposite trend in the mitochondrial dinucleotide contents, as well as the different respiratory efficiency, of the two types of mutants are in line with those detected during exponential growth on ethanol (Agrimi et al., 2011).

**Table 2:** Respiratory parameters determined for *NDT1*-over *and ndt1*Δ *ndt2*Δ strains.

**Genetic Background**	**Strain**		**J**_**R**_					**J**_**MAX**_			
		**Exp**	**Day 0**	**Day 1**	**Day 2**	**Day 3**	**Exp**	**Day 0**	**Day 1**	**Day 2**	**Day 3**
	wt	**7.12** ± 0.29	**11.23** ± 0.29	**14.24** ± 0.32	**10.63** ± 0.16	**7.59** ± 0.12	**17.32** ± 0.32	**21.76** ± 0.59	**27.65** ± 0.13	**25.11** ± 0.26	**21.31** ± 0.31
CEN-PK 113-7D	*NDT1*-over	**7.28** ± 0.31	**11.98** ± 0.17	**13.88** ± 0.32	**10.25** ± 0.24	**7.38** ± 0.17	**17.48** ± 0.21	26.29^**^± 0.19	30.15^**^± 0.37	28.26^**^± 0.25	23.67^**^± 0.31
	*ndt1Δndt2Δ*	**7.09** ± 0.22	9.66^**^± 0.41	10.77^**^± 0.27	8.52^**^± 0.17	6.53^**^± 0.26	**16.76** ± 0.16	18.12^**^± 013	21.35^**^± 0.47	19.57^**^± 0.14	17.73^**^± 0.09
W303-1A	wt	**7.76** ± 0.07	**9.47** ± 0.13	**14.41** ± 0.25	**11.69** ± 0.15	**7.52** ± 0.21	**16.23** ± 0.37	**19.97** ± 0.27	**26.33** ± 0.26	**25.41** ± 0.17	**24.28** ± 0.24
	*ndt1Δndt2Δ*	**7.55** ± 0.35	8.42^**^± 0.17	11.83^**^± 0.28	9.64^**^± 0.36	6.38^**^± 0.24	**15.97** ± 0.25	17.34^*^± 0.27	20.13^**^± 0.19	18.99^**^± 0.31	16.54^**^± 0.26
**Genetic Background**	**Strain**		**J**_**TET**_					**net**_**R**_			
		**Exp**	**Day 0**	**Day 1**	**Day 2**	**Day 3**	**Exp**	**Day 0**	**Day 1**	**Day 2**	**Day 3**
	wt	**1.43** ± 0.41	**2.13** ± 0.19	**2.80** ± 0.45	**3.30** ± 0.29	**3.89** ± 0.19	**5.32** ± 0.27	**9.12** ± 0.16	**11.44** ± 0.26	**7.26** ± 0.16	**3.69** ± 0.23
CEN-PK 113-7D	*NDT1*-over	**1.72** ± 0.09	3.47^**^± 0.26	3.97^**^± 0.27	4.22^**^± 0.37	5.58^**^± 0.33	**5.55** ± 0.09	**8.51** ± 0.17	**9.91** ± 0.32	5.93^**^± 0.24	1.82^**^± 0.17
	*ndt1Δndt2Δ*	**1.24** ± 0.38	1.31^**^± 0.12	1.43^**^± 0.38	1.51^**^± 0.18	1.84^**^± 0.26	**5.61** ± 0.13	**8.35** ± 026	**9.37** ± 0.16	**7.12** ± 0.17	4.73^**^± 0.26
W303-1A	wt	**1.24** ± 0.38	**1.58** ± 0.38	**3.23** ± 0.44	**3.55** ± 0.22	**3.98** ± 0.19	**6.52** ± 0.23	**7.87** ± 0.26	**11.18** ± 0.11	**8.14** ± 0.31	**3.54** ± 0.13
	*ndt1Δndt2Δ*	**1.04** ± 0.26	1.18^**^± 0.09	1.27^**^± 0. 32	1.46^**^± 0.29	1.59^**^± 0.27	**6.14** ± 0.19	**7.23** ± 0.08	10.59^*^± 0.14	**8.06** ± 0.23	4.53^*^± 0.21

Oxygen consumption rates (J) are expressed as pmol/10^6^ cells/s. Basal respiration rate (J_R_), uncoupled respiration rate (J_MAX_), non-phosphorylating respiration (J_TET_), and net respiration (net_R_ = J_R_-J_TET_). Substrates and inhibitors used in the measurements of the respiratory parameters are detailed in the text. Exp, exponential growth phase; Day 0, diauxic shift. Data refer to mean values determined in three independent experiments with three technical replicates each. SD is indicated. Values obtained for wt were used as reference for comparisons with NDT1-over and ndt1Δndt2Δ cells. (

* P ≤ 0.05 and

** *P ≤ 0.01, one-way ANOVA test). Mean values are provided in bold*.

To the best of our knowledge, Ndt1 and Ndt2 are the only mitochondrial NAD^+^ carriers described so far in *S.cerevisiae* (Todisco et al., 2006) and their transport activity for NAD^+^ is also consistent with the cellular localization of the enzymes involved in NAD^+^ biosynthesis, which are outside the mitochondria, and with the lack of NAD^+^- synthesizing enzymes in the yeast mitochondria (Kato and Lin, 2014). However, since in the mitochondria of the *ndt1*Δ*ndt2*Δ mutant, NAD^+^ is present, albeit at low levels, it cannot be excluded that this dinucleotide can be imported in the mitochondria with lower efficiency by other carrier systems. Indeed, many mitochondrial transporters often exhibit some overlapping of the transported substrates (Palmieri et al., 2006). In addition, other systems contribute to the homeostasis of the intramitochondrial NAD pool, as well as to balance dinucleotide pools between mitochondria and cytosol/nucleus. They include, among others, two NADH dehydrogenases (Nde1 and Nde2) distributed on the external surface of the inner mitochondrial membrane and the glycerol-3-phosphate shuttle (Bakker et al., 2001). Nde1 and Nde2 directly catalyze the transfer of electrons from cytosolic NADH to ubiquinone without the translocation of protons across the membrane. In such a way, the ETC is supplied with electrons (Baccolo et al., 2018). The expression of *NDE1* and *NDE2* is induced after the diauxic shift (Bakker et al., 2001). With regard to the glycerol-3-phosphate shuttle, it is a system of crucial importance under conditions where the availability of energy is limited (Rigoulet et al., 2004). In the glycerol-3-phosphate shuttle, cytosolic glycerol-3-phosphate dehydrogenase oxidizes cytosolic NADH catalyzing the reduction of dihydroxyacetone phosphate to glycerol-3-phosphate. Subsequently, into the mitochondrial matrix, glycerol-3-phosphate delivers its electrons to ubiquinone via the FAD-dependent glycerol-3-phosphate dehydrogenase, Gut2 (Bakker et al., 2001). The result is a stepwise transfer of electrons from the cytosol to the respiratory chain. Consequently, despite the low mitochondrial dinucleotide contents, the *ndt1*Δ*ndt2*Δ mutant might feed the oxidative phosphorylation with the NADH produced in the cytosol.

Afterward, given the differences in the state of respiration, we decided to analyze the content of superoxide anion (${\text{O}}_{2}^{-}$), which is the primary mitochondrial reactive oxygen species (ROS) produced by electron leakage from the respiratory chain. It is known that ${\text{O}}_{2}^{-}$/ROS accumulation limits the long-term survival of yeast cells during CLS (Pan, 2011; Breitenbach et al., 2014; Baccolo et al., 2018). In the *ndt1*Δ*ndt2*Δ chronologically cells and in the *NDT1*-over ones, a strong decrease and increase in ${\text{O}}_{2}^{-}$ content was observed, respectively, compared to that of the wt (Figure 5C) consistent with non-phosphorylating respiration data (Table 2). Indeed, it is a state of non-phosphorylating respiration prone to generate ${\text{O}}_{2}^{-}$ (Hlavata et al., 2003; Guerrero-Castillo et al., 2011). In addition, we analyzed the mitochondrial functionality by measuring the IRC, which defines the percentage of viable cells competent to respire (Parrella and Longo, 2008). Starting from the diauxic shift where all the strains were respiration-competent, a different trend of the IRC was observed for the *ndt1*Δ*ndt2*Δ strain and in the *NDT1*-over one. In the former, a lower decrease in the mitochondrial functionality was detected and at Day 18 the IRC was still about 60% against about 40% in the wt (Figure 5D). In the *NDT1*-over chronologically aging cells, a dramatic time-dependent loss of mitochondrial functionality was observed reaching at Day 18 values close to zero (Figure 5D). This is in line with the increased ${\text{O}}_{2}^{-}$ formation because it is known that ROS levels influence mitochondrial fitness and mitochondrial dysfunctions, in turn, lead to a higher propensity to produce ROS (Breitenbach et al., 2014).

**Figure 6:**
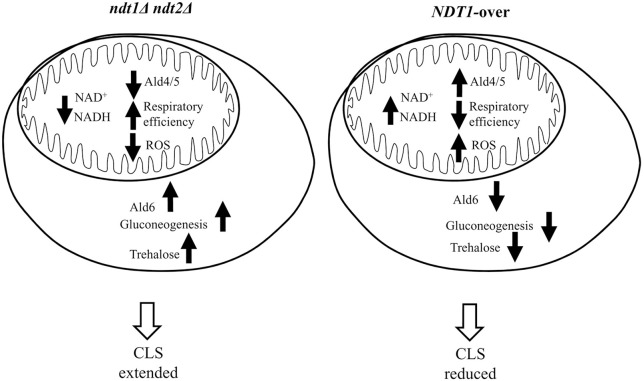
Simplified scheme summarizing the effects of the altered expression of mitochondrial NAD^+^ carriers during chronological aging.

Thus, taken together all the results clearly indicate that, in the context of a standard CLS experiment, alterations in the expression of the specific mitochondrial NAD^+^ carriers determined by *NDT1* and *NDT2* double deletion and *NDT1* overexpression deeply influence the metabolism with opposite outcomes on chronological longevity (Figure 6). We found that the former extends CLS, whereas the latter shortens it. This is a direct consequence, on the one hand, of the participation of NAD^+^ together with its reduced counterpart, NADH, in a wide range of metabolic reactions modulating the activity of compartment-specific pathways among which the TCA cycle and the ETC in the mitochondria and the glycolysis/gluconeogenesis in the cytosol. On the other hand, the CLS is regulated by signaling pathways that coordinate the metabolic reprogramming required to ensure longevity (Breitenbach et al., 2014; Zhang and Cao, 2017). On the whole, in the *ndt1*Δ*ndt2*Δ chronologically aging cells and in the *NDT1*-over ones an opposite metabolic remodeling is observed, involving both cytosolic (gluconeogenesis), and mitochondrial (TCA and respiration) metabolic pathways (Figure 6), which are operative during chronological aging. Lack of the mitochondrial NAD^+^ carriers results in a reduced oxygen consumption that does not depend upon dysfunctional mitochondria but most likely upon a decreased amount of reducing equivalents provided by a TCA cycle, the activity of which is reduced. Nevertheless, this mutant maintains a net respiration close to that of the wt indicating that in the mutant the respiration, albeit reduced, is more efficient. This confirms previous data on *ndt1*Δ*ndt2*Δ cells exponentially growing on ethanol that show a better coupling of respiration and phosphorylation (Agrimi et al., 2011). Such a state of more coupled respiration is less prone to generate hazardous ${\text{O}}_{2}^{-}$ decreasing the risk of inducing oxidative stress and its detrimental effects on cell survival of non-dividing cells during chronological aging: in agreement with this, *ndt1*Δ*ndt2*Δ cells are long-lived. In agreement with a short-lived phenotype accompanied by ${\text{O}}_{2}^{-}$ accumulation and severe mitochondrial damage, *NDT1*-over chronologically aging cells display an enhanced uncoupled respiration and a lower respiratory efficiency. As in the case of the *ndt1*Δ*ndt2*Δ cells, changes in the state of respiration have been already observed in *NDT1*-over cells exponentially growing on ethanol (Agrimi et al., 2011). In this context of fully respiratory metabolism, the *NDT1* overexpression determines a decrease in the respiratory efficiency similar to that described here when cells have exhausted glucose and shift to ethanol-driven respiration. These results further underline how the mitochondrial NAD^+^ carriers and, consequently, the availability of mitochondrial NAD^+^, and/or NADH is important to achieve an efficient respiration and how this aspect can influence the CLS.

Concerning gluconeogenesis, the enzymatic activity of Pck1 is generally considered the main flux-controlling step in the pathway. The gluconeogenic activity of this enzyme depends on its de/acetylation state (Lin et al., 2009; Casatta et al., 2013). Indeed, an increase in the enzymatic activity of Pck1 correlates with an increase in its acetylated active form promoting gluconeogenesis and CLS (Lin et al., 2009; Casatta et al., 2013; Orlandi et al., 2017a,b). The enzyme responsible for Pck1 deacetylation (inactive form) is the NAD^+^-dependent deacetylase Sir2 (Lin et al., 2009). During chronological aging, lack of Sir2 correlates with an increase of the acetylated Pck1 and with a carbohydrate metabolism shift toward glyoxylate-requiring gluconeogenesis increasing CLS (Casatta et al., 2013; Orlandi et al., 2017a,b). It is conceivable that, as the deacetylase activity of Sir2 relies on NAD^+^, the low level of this dinucleotide in the *ndt1*Δ*ndt2*Δ mutant might decrease Sir2-mediated deacetylation of Pck1 and consequently increase gluconeogenesis and CLS. Differently, in the *NDT1*-over mutant, a different availability of NAD^+^ might favor Sir2 enzymatic activity leading to an increase of the deacetylated inactive form of Pck1 and to the observed decrease of gluconeogenesis and CLS.

Furthermore, in the *ndt1*Δ*ndt2*Δ mutant and the *NDT1*-over one, other metabolic traits that result from an enhancement and a down-regulation, respectively, of the cytosolic Ald6/glyoxylate/gluconeogenesis axis fit-well with their CLS. Indeed, Ald6 activity requires NADP^+^ providing NADPH, which is also provided by the pentose phosphate pathway fueled by the gluconeogenesis with glucose-6 phosphate. NADPH is a source of reducing energy and an essential cofactor for glutathione/thioredoxin-dependent enzymes that are essential for protecting cells from oxidative stress (Pollak et al., 2007). Thus, NADPH availability can contribute to influence the physiological state of the cells and consequently their survival. In this context, the *ndt1*Δ*ndt2*Δ mutant might be further favored by an enhanced gluconeogenic activity that leading also to increased intracellular trehalose stores, ensures viability during chronological aging. On the contrary the down-regulation of the Ald6/glyoxylate/gluconeogenesis axis observed in the *NDT1*-over mutant decreasing cellular protection systems, might contribute to affect negatively the CLS.

To date, substantial number of evidence points out that lowering NAD^+^ levels can decrease Sirtuin activities and affect the aging process both in *S.cerevisiae* and mammalian cells (Imai and Guarente, 2016). In particular, in yeast lack of the nicotinic acid phosphoribosyltransferase, Npt1, which in the salvage pathway generates NAD^+^ from nicotinic acid (NA), reduces NAD^+^ content. This is accompanied by loss of silencing and decrease in RLS (Smith et al., 2000), as NAD^+^ levels are not sufficient for Sir2 to function (Ondracek et al., 2017). Addition of nicotinamide riboside (an NAD^+^ precursor) corrects the deficit in NAD^+^ content of the *npt1*Δ mutant, promotes Sir2-dependent silencing and extends RLS (Belenky et al., 2007). Furthermore, yeast cells grown in media lacking NA has a short RLS and low NAD^+^ levels; supplementation of isonicotinamide extends RLS in a Sir2-dependent manner by restoring NAD^+^ content and alleviating the nicotinamide (NAM) inhibition on Sir2 (McClure et al., 2012). Indeed, NAM is an NAD^+^ precursor that is also an endogenous non-competitive inhibitor of Sir2 (Sauve et al., 2005). Yeast cells grown in the presence of NAM have the same phenotype of *sir2*Δ ones such as silencing defects and a short RLS (Sauve et al., 2005). In the context of chronological aging, NAM supplementation at the diauxic shift results in a phenocopy of chronologically aging *sir2*Δ cells: due to the inhibition of Sir2, Pck1 enzymatic activity, and gluconeogenesis are promoted and CLS is extended (Orlandi et al., 2017a). On the opposite, resveratrol, a Sirtuin activating compound, restricts CLS by enhancing Sir2 activity, in particular Sir2-mediated deacetylation of Pck1, and consequently gluconeogenesis is decreased (Orlandi et al., 2017b).

In conclusion, taken together all our results show that affecting the cellular distribution and the content of NAD^+^ has a deep impact on both metabolism and chronological aging and that a critical functional role is played by the Sir2 activity. In addition, our data indicate that in order to elucidate the intimate interplay between NAD^+^, Sirtuins and aging, it will be important to determine how NAD^+^ levels change in different compartments during aging and the tissue-specific regulation of NAD metabolism and Sirtuin activity.

## Author Contributions

MV conceived the project. MV and IO designed the experiments. IO and GS performed the experiments. MV wrote the manuscript. All authors have read and approved the final version of the manuscript.

### Conflict of Interest Statement

The authors declare that the research was conducted in the absence of any commercial or financial relationships that could be construed as a potential conflict of interest.
